# A cross‐sectional questionnaire survey involving physicians for the clarification of the diagnosis and current status of therapeutic intervention of psoriatic arthritis in Japan

**DOI:** 10.1111/1346-8138.17055

**Published:** 2023-12-12

**Authors:** Akihiko Asahina, Yukie Minami, Hideto Kameda

**Affiliations:** ^1^ Department of Dermatology The Jikei University School of Medicine Tokyo Japan; ^2^ Medical Affairs Department Maruho Co., Ltd. Osaka Japan; ^3^ Division of Rheumatology, Department of Internal Medicine, Faculty of Medicine Toho University Tokyo Japan

**Keywords:** diagnosis, psoriasis, psoriatic arthritis, screening, therapeutic intervention

## Abstract

Patients with psoriatic arthritis (PsA) often experience comorbid, irreversible joint destruction, therefore early diagnosis and treatment of PsA are important. The diagnosis requires a comprehensive assessment, which includes an interview, a physical examination, a visual examination of the skin and nails, a blood test, and an imaging test. To clarify how patients with PsA are actually diagnosed and how physicians collaborate among clinical departments, we conducted a web‐based questionnaire survey of 500 physicians (dermatologists, rheumatologists, and orthopedists) frequently involved in PsA treatment in Japan. The survey showed that those patients are rarely confirmed to have axial arthritis, peripheral arthritis, enthesitis, or dactylitis by general dermatology practitioners (GP dermatologists). Overall, <60% of patients suspected of having PsA underwent PsA examination by GP dermatologists more than once every 6 months; this percentage is lower than that of patients who underwent PsA examination by rheumatologists and orthopedists. The Psoriatic Arthritis Screening and Evaluation (PASE) questionnaire is the most commonly used for PsA screening. However, users of PASE were only 11.0%, 25.3%, 14.8%, and 24.1% of GP dermatologists, attending dermatologists in hospitals (HP dermatologists), rheumatologists, and orthopedists, respectively. While >80% of HP dermatologists, rheumatologists, and orthopedists used imaging tests (ultrasound, X‐ray, and magnetic resonance imaging) for PsA screening, only 40% of GP dermatologists performed imaging tests. Regarding the demands on the healthcare environment of PsA treatment, early diagnosis and treatment for PsA are crucial in every clinical department. The present study showed that GP dermatologists rarely perform imaging tests or confirm a PsA diagnosis, thus patients may miss out on appropriate treatment through collaboration among clinical departments and step‐up therapy. Because patients with PsA present diverse comorbid clinical symptoms, early diagnosis, including routine imaging tests, and appropriate treatment in collaboration with other experts are necessary.

## 1 INTRODUCTION

Psoriatic arthritis (PsA) is characterized by various clinical symptoms, including skin psoriasis, peripheral arthritis in the distal interphalangeal joints, and joint symptoms of axillary arthritis similar to sacroiliac arthritis. Additionally, nail psoriasis, enthesitis, and dactylitis are seen in approximately 60%, 30%, and 25% of patients, respectively,^1^ therefore early diagnosis and treatment of PsA are important and should be based on a comprehensive assessment that includes a physical examination, a visual examination of the skin and nails, an interview, a blood test, and an imaging test.^2^ The prevalence of PsA in Japan has increased in recent years as a result of westernization of dietary habits, widespread public awareness of the disease, and improvements in diagnostic methods.^3^ PsA in Japan accounts for 1.9%, 14.3%, and 15.3% of all psoriasis cases according to the health insurance claims database of the Japan Medical Data Center,^4^ a report from rheumatologists,^3^ and a report from dermatologists,^5^ respectively.

However, PsA has a wide range of clinical symptoms, making its diagnosis difficult. Dermatologists often examine skin psoriasis owing to their expertise, whereas patients with joint symptoms are more likely to consult orthopedists and/or rheumatologists. The stance on PsA screening might differ between dermatology clinics and hospitals depending on the circumstances of the facilities, therefore collaboration among different clinical departments becomes important. Because cutaneous lesions appear before other symptoms in most patients with PsA,^6^ dermatologists play an important role in the early recognition and diagnosis of PsA. According to Spanish experts, the joint symptoms of psoriasis patients should be confirmed ideally within 6 months or at least within a year.^7^ However, the 6‐month lag between the onset of PsA symptoms and the first diagnosis increases the risk of bone erosion and dysfunction.^8^ Furthermore, the European League Against Rheumatism (EULAR) treatment guidelines recommend following a flow chart to help achieve the desired therapeutic effect within 3–6 months and to indicate the need to step up therapy if the therapeutic goals are not met.^9^ For the appropriate treatment step‐up, a routine examination of joint symptoms is necessary. However, the medical care that should be provided to patients who have or are at risk of developing PsA is not well defined. In this study, we conducted a web‐based questionnaire survey on dermatologists, as well as rheumatologists and orthopedists who are commonly involved in PsA treatment to understand the actual status of PsA treatment and collaboration among clinical departments.

## 2 METHODS

We conducted an online questionnaire survey in Japan on a panel of physicians at Plamed Inc. who met the following conditions: dermatologists who had treated at least five patients with psoriasis vulgaris or PsA in the last 3 months; physicians who treated patients as rheumatologists and had treated at least two patients with PsA in the last 3 months; and physicians who treated patients as orthopedists and had treated at least two patients with PsA in the last 3 months. The questionnaire was sent to 1850 dermatologists, 569 rheumatologists, and 3790 orthopedists.

The survey included questions about the following criteria (Supporting Information Table S1): (1) management style (clinic or hospital); (2) number of patients with psoriasis vulgaris and PsA treated in the last 3 months and the severity of the disease; (3) facilities certified for biologics use by the Japanese Dermatological Association (for dermatologists only); (4) number of patients suspected of having PsA and reason for their visit; (5) action taken for patients suspected of having PsA; (6) action taken for patients with confirmed diagnoses of PsA; (7) frequency of confirming each symptom (skin psoriasis and nail psoriasis, axial arthritis, peripheral arthritis, enthesitis, and dactylitis) for patients with psoriasis vulgaris, patients suspected of having PsA, and patients with confirmed diagnoses of PsA; (8) knowledge of various PsA questionnaires (Psoriatic Arthritis Screening and Evaluation^10^ [PASE], Psoriasis Epidemiology Screening Tool^11^ [PEST], Japanese version of the early psoriatic arthritis screening^12^ [J‐EARP], Toronto Psoriatic Arthritis Screen^13^ [ToPAS], and Psoriasis and Arthritis Screening Questionnaire^14^ [PASQ]); (9) implementation of PsA screening methods (questionnaire, blood test, and imaging); and (10) reason for performing or not performing PsA screening (severity, nail psoriasis, etc.).

This survey was approved by the ethics committee of the Kitamachi Clinic (Tokyo, Japan; approval number: 11001110) and performed according to the ethical principles stipulated in the Declaration of Helsinki and Ethical Guidelines for Medical and Health Research Involving Human Subjects.

## 3 RESULTS

The questionnaire survey was conducted from February 18, 2022, to February 25, 2022. The responses were received from 325 dermatologists (127 general practitioners and 198 attending physicians), 88 rheumatologists, and 87 orthopedists. For tally purposes, dermatologists were divided into clinic and hospital groups according to their management styles. The background characteristics of the respondents from each clinical department are shown in Table 1.

**Table 1:** Background information of the respondents.

			GP dermatologists, *n* = 127	HP dermatologists, *n* = 198	Rheumatologists, *n* = 88	Orthopedists, *n* = 87
Management style	Clinic, *n*	%	100	—	6.8	18.4
	Hospital, *n*	%	—	100	93.2	81.6
Facilities certified for biologics use by The Japanese Dermatological Association	Yes (%)	%	20.5	85.4	—	—
	No (%)	%	79.5	14.6	—	—
Number of patients in the last 3 months	PsV		28.8	35.6	2.2	1.7
	PsA‐suspected patients		1.9	4.6	4.4	4.6
	PsA		1.3	5.9	9.1	5.5
Severity of psoriasis vulgaris (BSA)	BSA less than 3% (%)	%	58.9	45.2	51.1	45.4
	BSA 3% to less than 10% (%)	%	31.6	36.2	40.0	33.6
	BSA 10% or more (%)	%	9.5	18.6	8.9	21.1
Average duration of examination^a^	PsV: initial visit (min)	minutes	11.9	17.0	17.8	19.3
	PsV: return visit (min)	minutes	5.5	7.1	8.3	8.7
	PsA: initial visit (min)	minutes	12.8	19.5	23.4	20.9
	PsA: return visit (min)	minutes	6.2	8.2	9.3	8.7

Abbreviations: BSA, body surface area; GP dermatologists, general dermatology practitioners; HP dermatologists, attending dermatologists in hospital; PsA, psoriatic arthritis; PsV, psoriasis vulgaris.

^a^ Responses of initial and return visits (*n*) of PsV were 39 for rheumatologists and 38 orthopedists.

### 3.1 History of patients with PsA and their reasons for visiting the doctor or facility

**Figure 1:**
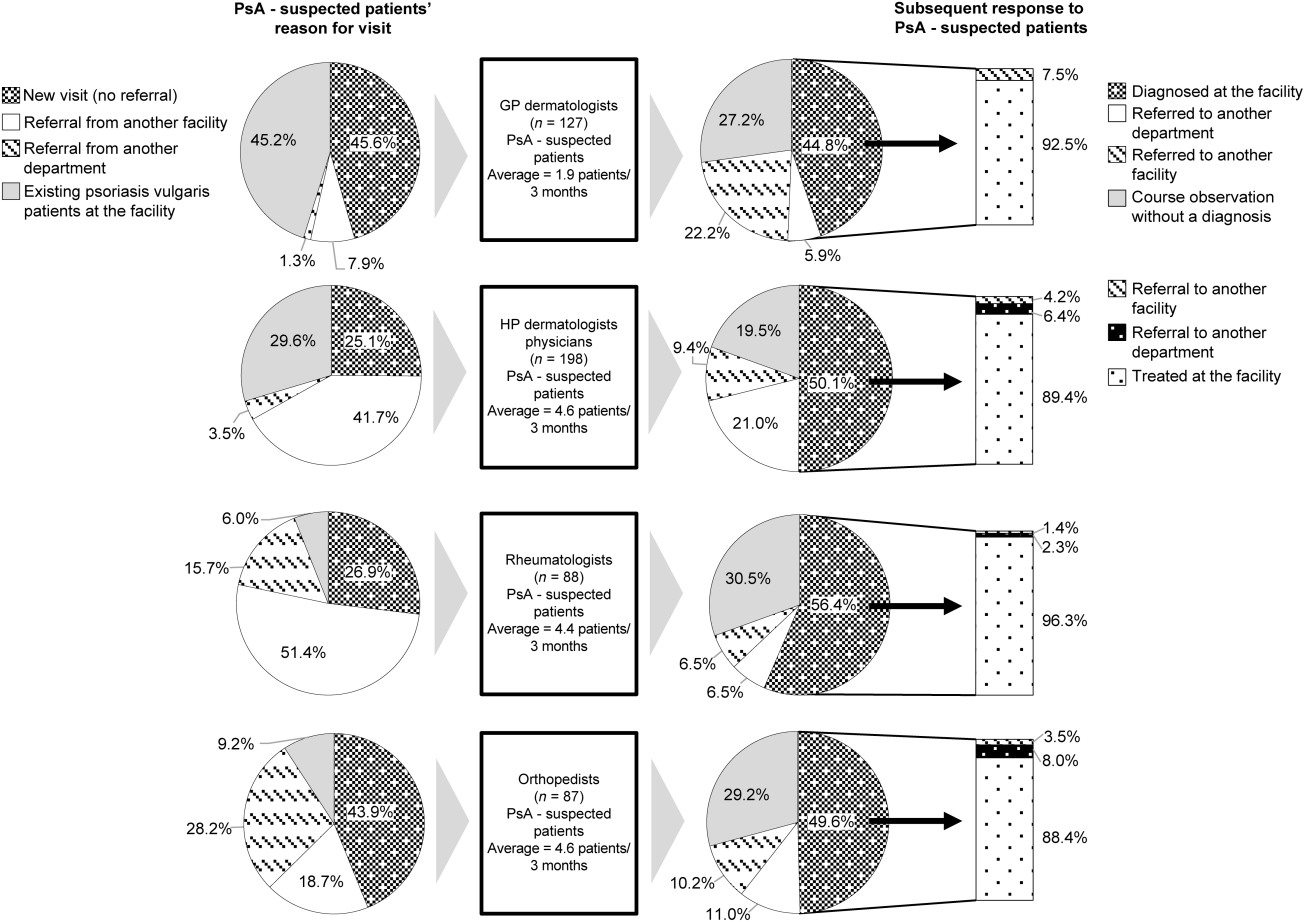
History of patients with psoriatic arthritis and their reasons for visiting the hospital. We surveyed the patients suspected of having PsA in the past 3 months. The pie graph shows the diagnostic response, whereas the bar graph shows the handling outcome. GP dermatologists, 127; HP dermatologists, 198; rheumatologists, 88; and orthopedists, 87. GP dermatologist, general dermatology practitioners; HP dermatologists, attending dermatologists in hospital; PsA, psoriatic arthritis.

In the history of patients with PsA and their reasons for visiting the hospital, for general dermatology practitioners (GP dermatologists) and orthopedists, the most common reason was patient visits on their own (Figure 1). Meanwhile, for attending dermatologists in hospitals (HP dermatologists) and rheumatologists, the most common reason was referrals from another facility or department. In the last 3 months, the numbers of patients suspected of having PsA were 1.9, 4.6, 4.4, and 4.6 for GP dermatologists, HP dermatologists, rheumatologists, and orthopedists, respectively. It was found that 90% of patients with confirmed diagnoses of PsA received PsA treatment at the visited facility. On the contrary, 27.2%, 19.5%, 30.5%, and 29.2% of patients attended by GP dermatologists, HP dermatologists, rheumatologists, and orthopedists, respectively, were being observed at the facility without a definitive diagnosis.

### 3.2 Examination

**Figure 2:**
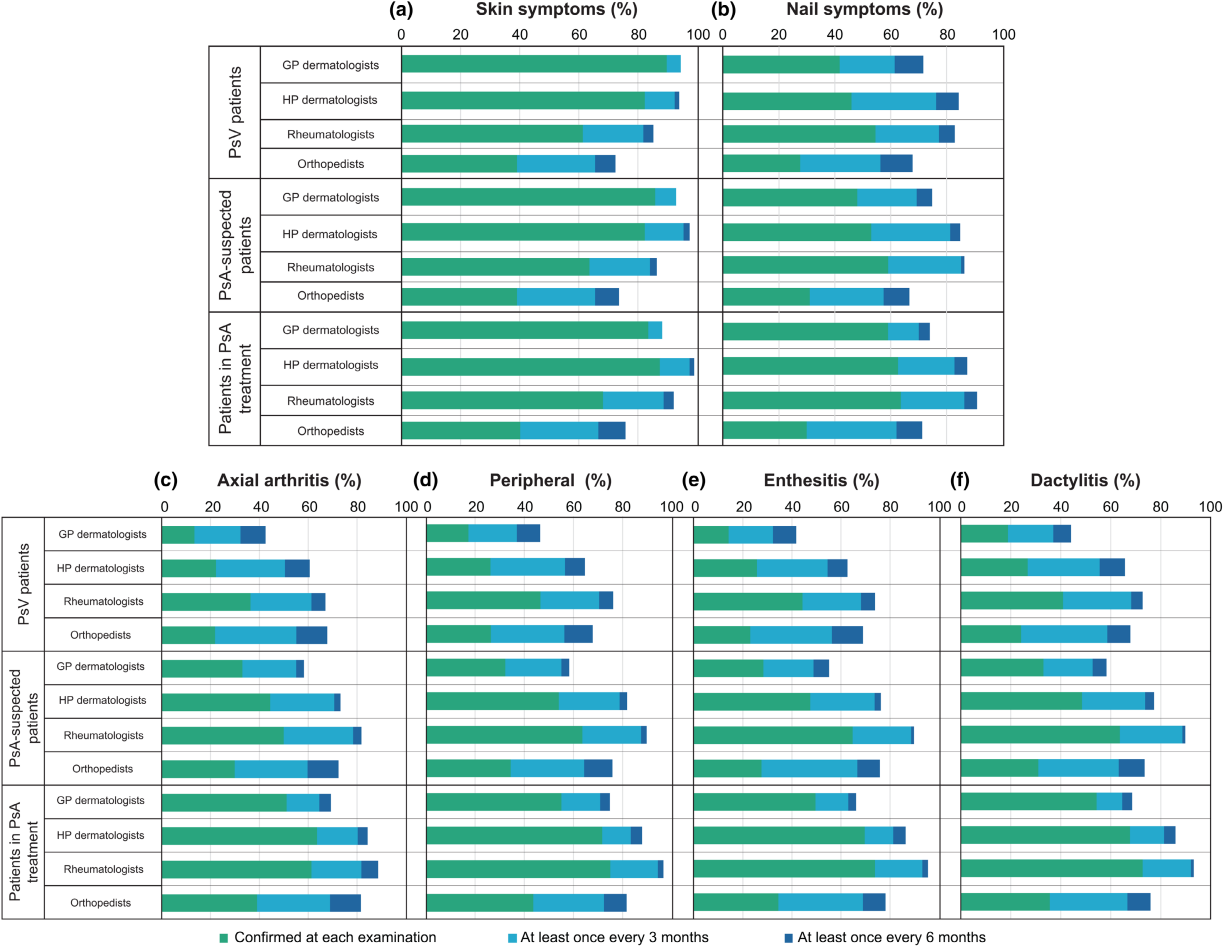
Frequency at which psoriatic arthritis symptoms are confirmed. (a) Skin psoriasis, (b) nail psoriasis, (c) axial arthritis, (d) peripheral arthritis, (e) enthesitis, and (f) dactylitis. GP dermatologists, 127; HP dermatologists, 198; rheumatologists, 88; and orthopedists, 87. GP dermatologists, general dermatology practitioners; HP dermatologists, attending dermatologists in hospital; PsA, psoriatic arthritis; PsV, psoriasis vulgaris.

Figure 2 shows the frequency of confirmation of skin psoriasis, nail psoriasis, and joint symptoms (axial arthritis, peripheral arthritis, enthesitis, and dactylitis) for patients with psoriasis vulgaris, patients suspected of PsA, and patients receiving PsA treatment. Patients suspected of having PsA were surveyed as patients with psoriasis vulgaris and musculoskeletal system symptoms. For patients who were suspected of having PsA and receiving PsA treatment, >70% of GP dermatologists, rheumatologists, and orthopedists confirmed joint symptoms at least once every 6 months, whereas <70% of GP dermatologists confirmed such symptoms, except for symptoms of peripheral arthritis in patients who were receiving PsA treatment. Rheumatologists and orthopedists often treat PsA joint symptoms, and fewer orthopedists than rheumatologists confirm peripheral arthritis, enthesitis, and dactylitis in patients suspected of having PsA and receiving PsA treatment.

**Figure 3:**
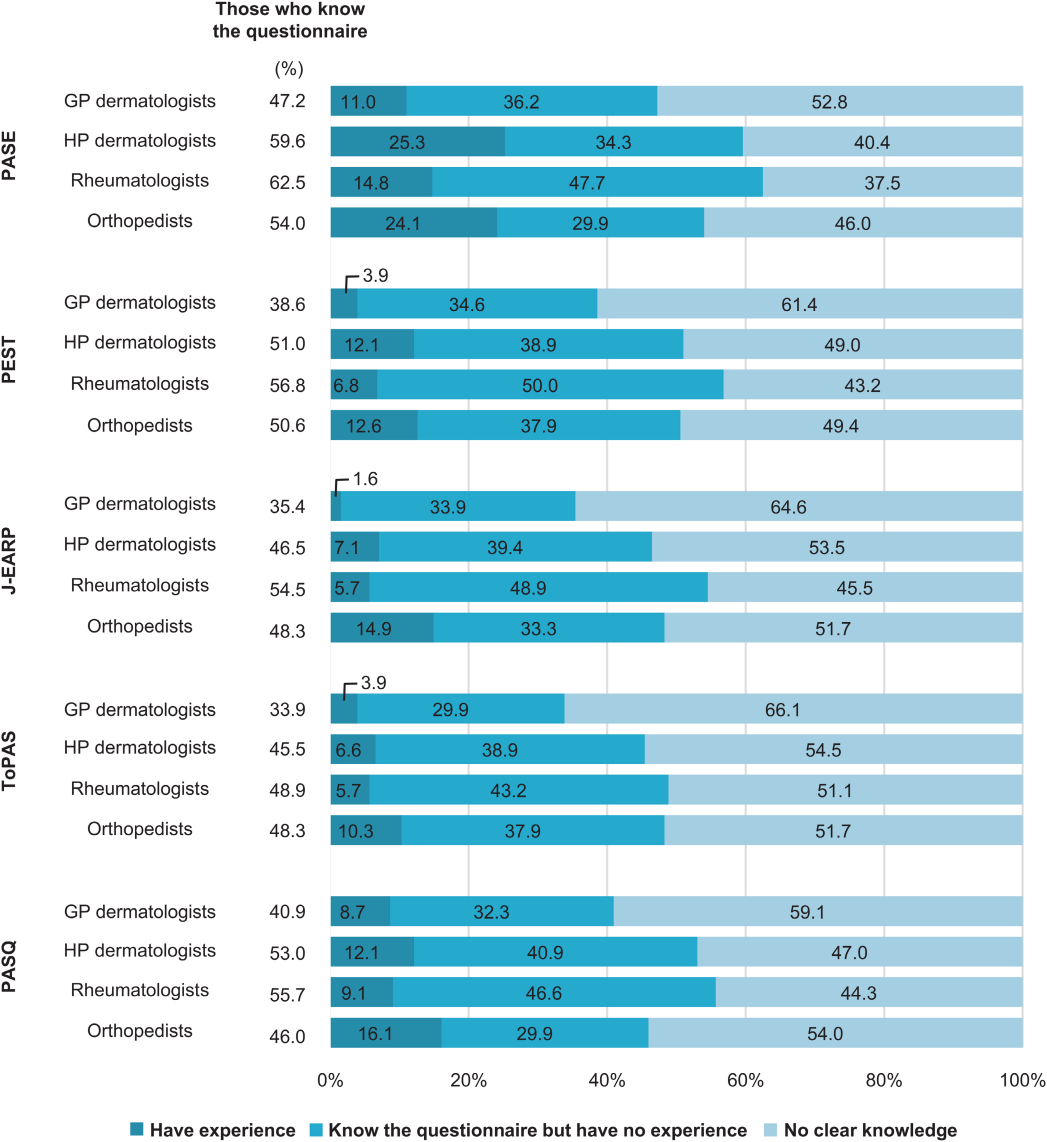
Knowledge of screening questionnaires and percentage of implementation. GP dermatologists, 127; HP dermatologists, 198; rheumatologists, 88; and orthopedists, 87. GP dermatologists, general dermatology practitioners; HP dermatologists, attending dermatologists in hospital; J‐EARP, Japanese version of the early psoriatic arthritis screening; PASQ, Psoriasis and Arthritis Screening Questionnaire; PEST, Psoriasis Epidemiology Screening Tool; ToPAS: Toronto Psoriatic Arthritis Screen.

The physicians' knowledge and implementation of screening questionnaires (PASE, PEST, J‐EARP, ToPAS, and PASQ) are shown in Figure 3. It was observed that half of the physicians knew how to apply these questionnaires, with an even lower rate of implementation. The implementation rates of the PASE questionnaire, which is well known and often used for the screening of PsA, were 11.0%, 25.3%, 14.8%, and 24.1% for GP dermatologists, HP dermatologists, rheumatologists, and orthopedists, respectively.

**Figure 4:**
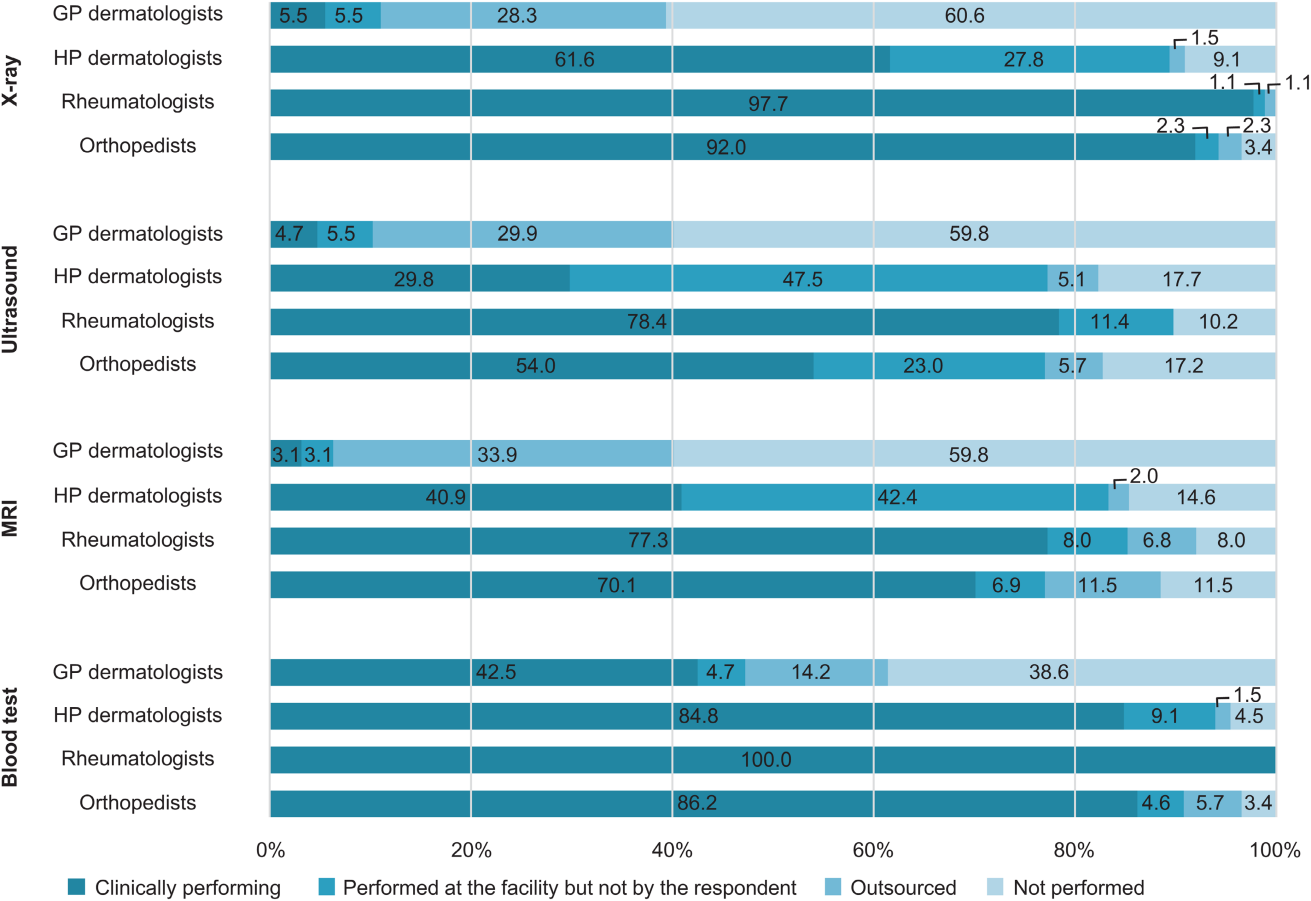
Percentage of imaging and blood tests performed. GP dermatologists, 127; HP dermatologists, 198; rheumatologists, 88; and orthopedists, 87. GP dermatologists, general dermatology practitioners; HP dermatologists, attending dermatologists in hospital; MRI, magnetic resonance imaging.

The frequency of the use of imaging techniques (X‐ray, ultrasound, and magnetic resonance imaging) and blood tests for PsA screening are shown in Figure 4. Imaging tests were performed by >80% of HP dermatologists, rheumatologists, and orthopedists when outsourced tests were included. In contrast, only 40% of GP dermatologists used imaging to screen patients.

We confirmed the reasons for imaging and/or blood tests with physicians who regularly perform them (Table 2). In all clinical departments, the tests were often conducted on “complaints of joint symptoms by patients,” which were followed by “joint symptoms discovered.” The response rates for “routine tests for psoriasis vulgaris patients” were 25.3%, 41.1%, 54.5%, and 37.2% for GP dermatologists, HP dermatologists, rheumatologists, and orthopedists, respectively.

**Table 2:** Reasons for psoriatic arthritis screening.

	GP dermatologists, *n* = 79^a^	HP dermatologists, *n* = 190^a^	Rheumatologists *n* = 88^a^	Orthopedists *n* = 86^a^
Complaints of joint symptoms by patients	75.9%	73.2%	67.0%	67.4%
Joint symptoms discovered by a physician	74.7%	69.5%	63.6%	67.4%
Nail psoriasis	45.6%	41.6%	42.0%	32.6%
Routine tests for PsV patients	25.3%	41.1%	54.5%	37.2%
Inflammatory response in a blood test	25.3%	30.5%	45.5%	41.9%
Family history of PsV or PsA	22.8%	21.1%	23.9%	19.8%
Certain comorbidity	20.3%	16.3%	23.9%	15.1%
Severe skin psoriasis	19.0%	19.5%	13.6%	10.5%
Long duration of PsV	17.7%	17.9%	12.5%	18.6%
Rash on the scalp, elbow, knee, or buttocks	15.2%	26.3%	25.0%	19.8%

Abbreviations: GP dermatologists, general dermatology practitioners; HP dermatologists, attending dermatologists in hospitals; PsA, psoriatic arthritis; PsV, psoriasis vulgaris.

^a^ Number of physicians who selected “clinically performing” “Performed at the facility but not by the respondent” or “outsourced” for imaging (X‐ray, ultrasound, and magnetic resonance imaging) and/or blood tests.

To establish the reasons for not performing a questionnaire, an imaging test, or a blood test for screening, we surveyed physicians who did not use these methods. Many physicians from all departments believed that physical findings and interviews were adequate for confirming the diagnosis (Supporting Information Table S2). Many GP dermatologists stated that they did not use imaging or blood tests owing to a lack of equipment and staff to manage these tests.

**Figure 5:**
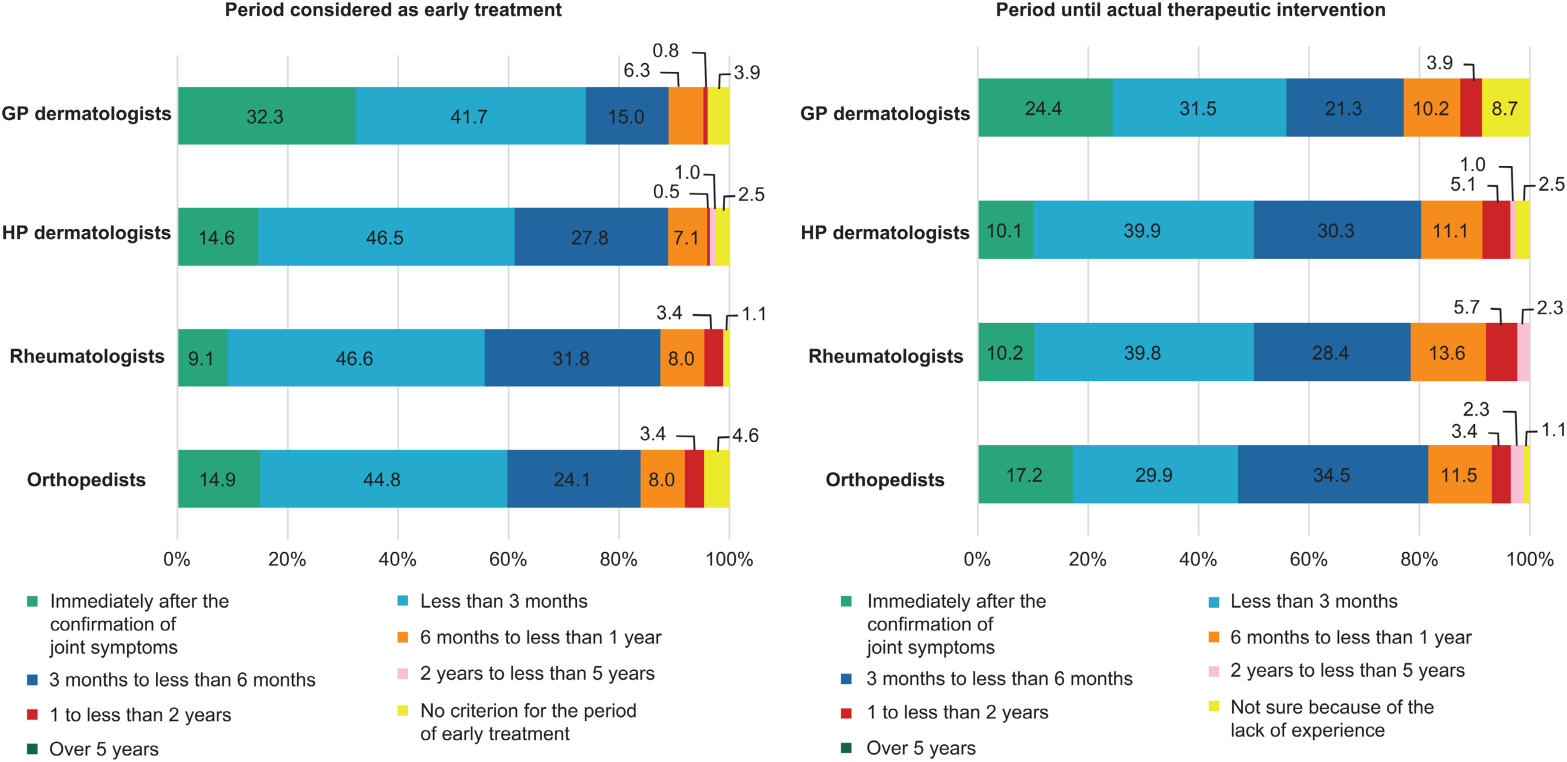
Percentage of period considered as early treatment and period until actual therapeutic intervention. GP dermatologists, 127; HP dermatologists, 198; rheumatologists, 88; and orthopedists, 87. GP dermatologists, general dermatology practitioners, HP dermatologists, attending dermatologists in hospital.

We inquired about the ideal or actual period between the confirmation of joint symptoms and the initiation of treatment (Figure 5). The period considered early treatment was <3 months (a sum of “immediately after the confirmation of joint symptoms” and “<3 months”) for 74.0%, 61.1%, 55.7%, and 59.8% of GP dermatologists, HP dermatologists, rheumatologists, and orthopedists, respectively. It was found that 32.3% of GP dermatologists considered “immediately after the confirmation of joint symptoms” to be early treatment. The period until the start of actual therapeutic intervention was <3 months (a sum of “immediately after confirmation of joint symptoms” and “<3 months”) for 55.9%, 50.0%, 50.0%, and 47.1% of GP dermatologists, HP dermatologists, rheumatologists, and orthopedists, respectively.

Regarding the demands for the healthcare environment for PsA treatment, >50% of physicians across all departments answered that “Medical collaboration that allows for early referral if psoriatic arthritis is suspected” and “Medical collaboration to ensure that patients receive the treatment they need as soon as possible” would be helpful in the better treatment of PsA (Table 3).

**Table 3:** Demands for psoriatic arthritis treatment.

	GP dermatologists, *n* = 127	HP dermatologists, *n* = 198	Rheumatologists, *n* = 88	Orthopedists, *n* = 87
Medical collaboration that allows for early referral if psoriatic arthritis is suspected	70.1%	64.1%	50.0%	55.2%
Medical collaboration to ensure that patients receive the treatment they need as soon as possible	57.5%	57.6%	51.1%	50.6%
To be able to make an early diagnosis without a specialist regarding psoriasis	24.4%	36.4%	27.3%	43.7%
Sharing information on patient progress after referral or acceptance	24.4%	24.2%	19.3%	20.7%
An opportunity to exchange opinions in cases where the diagnosis of psoriatic arthritis is not clear	22.0%	42.9%	34.1%	35.6%
An opportunity to exchange opinions on the treatment strategies and therapeutic options in case psoriatic arthritis is not clear	20.5%	37.9%	39.8%	32.2%
No specific demand	7.1%	3.5%	3.4%	5.7%

Abbreviations: GP dermatologists, general dermatology practitioners; HP dermatologists, attending dermatologists in hospital.

## 4 DISCUSSION

Considering that PsA may be accompanied by irreversible joint destruction, early diagnosis and treatment are vital. Dermatologists play a key role in the early diagnosis and treatment of patients with PsA because PsA follows skin psoriasis in most cases.^6^ Among PsA‐suspected patients who were examined in the last 3 months, 27.2% were examined by GP dermatologists without a confirmed diagnosis, and the same trend was observed by HP dermatologists, rheumatologists, and orthopedists. However, given that GP dermatologists do not often confirm joint symptoms or perform imaging tests, it is possible that they follow up with patients without imaging or blood tests. If dermatologists provide biologics to treat patients with PsA in Japan, the facility must be certified by the local academic society, therefore a conventional synthetic disease‐modifying antirheumatic drug (csDMARD) or apremilast is often prescribed as the initial treatment drug. However, apremilast's effectiveness in preventing joint destruction and axial arthritis is undetermined. If a csDMARD or apremilast is ineffective, a prompt step up to biologics is required for the treatment of PsA, therefore routine joint symptom confirmation is necessary for patients receiving PsA treatment. It was observed that many patients were referred from other departments or facilities to the rheumatology department, where their symptoms were frequently confirmed by conducting routine imaging tests. This can be attributed to the difficulty of definitively diagnosing patients with PsA without these tests. Orthopedists treat PsA‐related joint symptoms with the same frequency as rheumatologists. However, orthopedists confirm peripheral arthritis, enthesitis, or dactylitis less frequently than rheumatologists. Although these symptoms are characteristic of PsA and important for its diagnosis, some orthopedists may be unaware of the pathology of PsA and fail to confirm these symptoms. This suggestion is supported by the results of a previous survey^15^ that showed low satisfaction with orthopedists as cooperating partners.

In the previous survey,^15^ half of the respondents stated that they noticed joint symptoms and diagnosed PsA while treating patients for skin psoriasis. Furthermore, 70% of those physicians said that patients had these symptoms before diagnosis, indicating that there are cases where PsA symptoms were overlooked by physicians. In the present survey, the most common reasons for performing PsA screening were “when patients have symptom complaints” and “when physicians discover symptoms.” Physicians may overlook the possibility that the patient has PsA because patients with psoriasis vulgaris are unaware of PsA and might not report any complaints.^16^ Many patients with PsA who visit an orthopedic practitioner do so voluntarily rather than through a referral from another hospital. Patients may be complaining about their pain to orthopedists without being aware of the link between psoriasis and joint symptoms. Patients with psoriasis, especially those with severe skin psoriasis or nail psoriasis, should be informed of PsA and instructed to contact their attending physician if they experience pain.

Several screening questionnaires are being developed as an examination support tool for GP dermatologists, and they can be simple and useful tools for PsA diagnosis. Regardless of their importance, screening questionnaires are not widely known. The most prominent PASE questionnaire is only known by 47.2%, 59.6%, 62.5%, and 54.0% of GP dermatologists, HP dermatologists, rheumatologists, and orthopedists, respectively. Moreover, such questionnaires for screening patients were used by only 11.0%, 25.3%, 14.8%, and 24.1% of GP dermatologists, HP dermatologists, rheumatologists, and orthopedists, respectively. The most common reason for not using a questionnaire was that the practitioner felt they could diagnose the disease based on physical findings and interviews (Supporting Information Table S1); however, PsA diagnosis can be difficult because of its diverse symptoms. Furthermore, arthritis associated with psoriasis can have comorbid osteoarthritis and gout in addition to PsA,^17^ therefore PsA‐suspected patients must be identified using a differential diagnosis based on imaging and blood tests in addition to an interview and a physical examination. If the PASE score is ≥37, a prompt referral to an expert will aid in the early diagnosis and treatment of PsA.^10^ Because imaging tests are often unavailable in dermatology clinics because of a lack of equipment, collaboration with other hospitals and the use of a questionnaire (patients responding to the questionnaire while they wait for their examination) will lead to early PsA diagnosis and treatment.

According to the 2015 EULAR recommendations^18^ for active PsA, treatment should begin within 3 months of the onset of PsA symptoms. In addition, according to 2019 EULAR recommendations,^9^ in cases of mono/oligoarthritis, when NSAIDs were ineffective, the time to initiate csDMARD treatment was reduced from 3–6 months to 4 weeks. Furthermore, because the prognosis worsens with certain dysfunctions, 6 months would be considered the delayed period between symptom onset and the first visit.^8^ In the present survey, 70% of GP dermatologists and 60% of other physicians answered that <3 months is considered early treatment (the sum of “immediately after the confirmation of joint symptoms” and “<3 months”). In addition, 32% of GP dermatologists considered “immediately after the confirmation of joint symptoms” to be early treatment. However, the frequency with which GP dermatologists confirm symptoms and perform screening tests is lower than that of other clinical departments, so it is considered inadequate. In all clinical departments, only half of the physicians stated that the period until the start of actual treatment was <3 months (the sum of “immediately after the confirmation of joint symptoms” and “<3 months”). In some cases, the treatment was delayed by ≥6 months. Of GP dermatologists, 24% responded with “immediately after the confirmation of joint symptoms.” This was a higher percentage than in other clinical departments. This may be attributed to the easier access to dermatology clinics and increase in the number of available oral medications that can be conveniently administered in dermatology clinics. If symptoms worsen while on oral medication, a step up to biologics is necessary. In Japan, the use of biologics for dermatological purposes is limited to certain facilities. If the patients' current treatment is inadequate or their symptoms are worsening, they should be promptly referred to a facility where biologics are available.

In the present survey, there was a strong demand for cooperation with other hospitals for the early diagnosis and treatment of PsA, and there was no notable difference among the clinical departments in terms of their opinion of early treatment. However, only a small percentage of GP dermatologists confirmed the joint symptoms and used imaging tests in the actual treatment. Some physicians may be unsure of the method to confirm PsA‐related joint symptoms, may be unable to find a referral for additional testing, or may lack clear criteria for referring patients to other facilities. As a result, it is necessary for different clinical departments to collaborate and use their equipment to manage diverse PsA symptoms. Building a cooperative system that is appropriate for each department's characteristics will lead to the provision of optimal medical care to patients.

In the questionnaire surveys of university physicians, a system of cooperation between clinical departments at a facility is relatively well established, and such cooperation leads to the optimal management of patients with PsA.^15^ However, cooperation between hospitals and clinics, especially between different clinical departments, is uncommon. This would make referring the patient to an appropriate facility difficult. For improved PsA treatment, the physicians involved must communicate with one another and establish a referral‐friendly environment. Because this study was a web‐based questionnaire survey of physicians registered in Plamed Inc., the distribution of background characteristics such as age may differ from that of physicians actually involved in PsA treatment. The number of samples collected per clinical department was small and data from treatment records were not collected, therefore the responses were the subjective opinions of physicians.

## 5 CONCLUSIONS

Early treatment of PsA is important. To achieve appropriate and optimal therapeutic interventions, it is crucial to regularly confirm joint symptoms. Our data showed that the frequencies of joint symptom confirmations and imaging tests by GP dermatologists were low, indicating a possible loss of treatment opportunities. Using a screening questionnaire and routinely performing imaging tests in cooperation with other experts should allow for early diagnosis and appropriate treatment of PsA.

## CONFLICT OF INTEREST STATEMENT

Akihiko Asahina received payments for lectures and research funding from Maruho. Hideto Kameda has no conflict of interest to declare. Yukie Minami is an employee of Maruho.

## Supporting information


Supporting Information Table S1.
