# Supplementary material for: A cross‐sectional questionnaire survey involving physicians for the clarification of the diagnosis and current status of therapeutic intervention of psoriatic arthritis in Japan
Source: J Dermatol. 2023 Dec 12;51(2):261–70. doi: 10.1111/1346-8138.17055 (PMC11483934; doi:10.1111/1346-8138.17055)
Supplement: Supplementary file 1 — Supporting Information Table S1. [file JDE-51--s001.docx]

Supporting information

Supplemental Table 1 Whole questionnaire item of the survey

| Survey Item  Number | Survey Question |
| --- | --- |
| Question 1 | What is your main medical department? |
| Response choice 1 | 1 Dermatological department  2 Rheumatology department  3 Orthopaedic department  4 Other medical departments |
| Question 2 | What management style does you work at? |
| Response choice 2 | 1 Medical Clinic  2 University Hospital  3 National Hospital Organization  4 General Hospital |
| Question 3 | Is the hospital where you work an approved facilities certified for biologics use by The Japanese Dermatological Association? |
| Response choice 3 | 1 Yes  2 No |
| Question 4 | Could you please tell us the number of patients according to types of psoriasis in the last 3 months? |
| Response choice 4 | 1 Psoriasis vulgaris: x patients/month  2 Psoriatic arthritis: x patients/month  3 Psoriatic erythroderma: x patients/month  4 Psoriasis guttata: x patients/month  5 Psoriasis pustulosa: x patients/month  6 psoriasis in children: x patients/month |
| Question 5 | Could you please inform us the number of patients with psoriasis vulgaris treated by you in the last 3 months, according to severity of the disease? |
| Response choice 5 | 1 Mild (Body Surface Area (BSA) <3%): x patients/month  2 Moderate (BSA 3% to less than 10%): x patients/month  3 Severe (BSA 10% or higher): x patients/month |
| Question 6 | Could you please tell us the number of patients whom you have suspected as psoriatic arthritis in the last 3 months? |
| Response choice 6 | x patients/3 months |
| Question 7 | Could you please tell us the number of patients who were suspected of having psoriatic arthritis by you in the last 3 months, according to the following categories about the background that patients visited to you? |
| Response choice 7 | 1 Referrals from other hospitals for diagnosis or therapy: x patients/3 months  2 Referrals for diagnosis and therapy from other departments within your hospital (Could you also tell us the specific department?): x patients/3 months  3 New direct visiting to your hospital: x patients/3 months  4 Patient with a history of psoriasis vulgaris treated by you: x patients/3 months |
| Question 8 | Please tell us the number of patients suspected of having psoriatic arthritis treated by you in the last 3 months, according to the following categories about your response. |
| Response choice 8 | 1 Patient diagnosed with psoriatic arthritis in your department: x patients/3 months  2 Patients referred for confirmed diagnosis to other departments in the hospital: x patients/3 months  3 Patients referred to other hospitals for confirmed diagnosis: x patients/3 months  4 Patients under observation in your department with no confirmed diagnosis: x patients/3 months |
| Question 9 | Could you please tell us the number of patients diagnosed with psoriatic arthritis in your department in the last 3 months, by each current medical status? |
| Response choice 9 | 1 Patients who continue to be under the treatment in your department: x patients/3 months  2 Patients referred to other departments in your hospital for treatment after confirmed diagnosis: x patients/3 months  3 Patients referred to other hospitals for treatment after confirmed diagnosis: x patients/3 months |
| Question 10 | How often do you check the following items 1-6 for symptoms with patients in your daily clinical practice? Please answer with the average frequency of checks. |
| Response choice 10 | \|  \| Per visit \| Once every 2 months \| Once every 3 months \| Once every 6 months \| Once a year \| unscheduled \| Not checking at all \| \| --- \| --- \| --- \| --- \| --- \| --- \| --- \| --- \| \| Skin symptoms \| 1 \| 2 \| 3 \| 4 \| 5 \| 6 \| 7 \| \| Nail symptoms \| 1 \| 2 \| 3 \| 4 \| 5 \| 6 \| 7 \| \| Axial arthritis \| 1 \| 2 \| 3 \| 4 \| 5 \| 6 \| 7 \| \| peripheral arthritis \| 1 \| 2 \| 3 \| 4 \| 5 \| 6 \| 7 \| \| Enthesitis \| 1 \| 2 \| 3 \| 4 \| 5 \| 6 \| 7 \| \| Dactylitis \| 1 \| 2 \| 3 \| 4 \| 5 \| 6 \| 7 \| |
| Question 11 | Could you please tell us about your clinical practice in administering each screening tool (questionnaire) to patients diagnosed with suspected psoriatic arthritis? |
| Response choice 11 | \|  \| I have used it. \| I know it, but have not used it. \| I do not know about the tool. \| \| --- \| --- \| --- \| --- \| \| PASE \| 1 \| 2 \| 3 \| \| PEST \| 1 \| 2 \| 3 \| \| J-EARP \| 1 \| 2 \| 3 \| \| ToPAS \| 1 \| 2 \| 3 \| \| PASQ \| 1 \| 2 \| 3 \| |
| Question 12 | For patients with suspected psoriatic arthritis, what screening methods other than the Psoriatic Arthritis Screening Tool (Questionnaire) are used? |
| Response choice 12 | \|  \| Have used it. \| Used in the hospital, but not used by myself. \| Have asked this examination to other facilities. \| Have not used it. \| \| --- \| --- \| --- \| --- \| --- \| \| X-ray \| 1 \| 2 \| 3 \| 4 \| \| Ultrasonic diagnosis \| 1 \| 2 \| 3 \| 4 \| \| MRI (Magnetic Resonance Imaging) \| 1 \| 2 \| 3 \| 4 \| \| Blood test \| 1 \| 2 \| 3 \| 4 \| \| Other tests (please specify) \| 1 \| 2 \| 3 \| 4 \| |
| Question 13 | Could you please tell us the implementation frequency about each of screening methods for patients suspected of having psoriatic arthritis? |
| Response choice 13 | \|  \| Per visit \| Once every 2 months \| Once every 3 months \| Once every 6 months \| Once a year \| unscheduled \| \| --- \| --- \| --- \| --- \| --- \| --- \| --- \| \| Psoriatic Arthritis Screening Tool  (Questionnaire) \| 1 \| 2 \| 3 \| 4 \| 5 \| 6 \| \| X-ray \| 1 \| 2 \| 3 \| 4 \| 5 \| 6 \| \| Ultrasonic diagnosis \| 1 \| 2 \| 3 \| 4 \| 5 \| 6 \| \| MRI  (Magnetic Resonance Imaging) \| 1 \| 2 \| 3 \| 4 \| 5 \| 6 \| \| Blood test \| 1 \| 2 \| 3 \| 4 \| 5 \| 6 \| \| Other tests (please specify) \| 1 \| 2 \| 3 \| 4 \| 5 \| 6 \| |
| Question 14 | What kind of background information makes you perform screening for psoriatic arthritis in patients with psoriasis vulgaris? (multiple answers allowed) |
| Response choice 14 | 1 Screening is practiced regardless of background history.  2 In case that patient has medical comorbidities such as metabolic syndrome, uveitis, and inflammatory bowel disease.  3 In case the patient complain of symptoms of arthritis pain, stiffness, or swelling.  4 In case you observe swelling or other symptoms in the joints.  5 In case psoriasis lesions are identified on the nails.  6 In case skin rash (psoriatic skin rash) is observed on the scalp, elbows, knees, or buttocks.  7 In case the area of the skin rash of psoriasis is large.  8 In case the duration of psoriasis vulgaris is long.  9 In case the patient has a family history of psoriasis vulgaris or psoriatic arthritis.  10 In case of patients with changes in inflammation-related factors on blood tests.  11 Other background history (please specify) |
| Question 15 | Could you please tell us why you do not perform screening for psoriatic arthritis? (multiple answers allowed) |
| Response choice 15 |  |
| \|  \| Due to being able to diagnose the disease by physical examination and medical interview. \| Due to being able to diagnose by other methods. \| Due to spending much effort and time to use this screening method for psoriatic arthritis. \| Due not to trusting this screening method for psoriatic arthritis. \| Due to lack of equipment and staff to screen for psoriatic arthritis. \| Other reason (please specify) \| \| --- \| --- \| --- \| --- \| --- \| --- \| --- \| \| Psoriatic Arthritis Screening Tool  (Questionnaire) \| 1 \| 2 \| 3 \| 4 \| 5 \| 6 \| \| X-ray \| 1 \| 2 \| 3 \| 4 \| 5 \| 6 \| \| Ultrasonic diagnosis \| 1 \| 2 \| 3 \| 4 \| 5 \| 6 \| \| MRI  (Magnetic Resonance Imaging) \| 1 \| 2 \| 3 \| 4 \| 5 \| 6 \| \| Blood test \| 1 \| 2 \| 3 \| 4 \| 5 \| 6 \| \| Other tests (please specify) \| 1 \| 2 \| 3 \| 4 \| 5 \| 6 \| | |
| Question 16 | How long do you consider the period from the diagnosis of joint symptoms (joint pain, swelling, etc.) in patients with psoriasis vulgaris to be the period of early treatment? |
| Response choice 16 | 1 At the time of confirmed joint symptoms  2 Less than 3 months  3 More than 3 months to less than 6 months  4 More than 6 months to less than 1 year  5 More than 1 year to less than 2 years  6 More than 2 years to less than 5 years  7 More than 5 years  8 There is no defined standard for the duration of early treatment. |
| Question 17 | How long is the average time from diagnosis of joint symptoms (joint pain, swelling, etc.) to the start of treatment in patients with psoriasis vulgaris? |
| Response choice 17 | 1 At the time of confirmed joint symptoms  2 Less than 3 months  3 More than 3 months to less than 6 months  4 More than 6 months to less than 1 year  5 More than 1 year to less than 2 years  6 More than 2 years to less than 5 years  7 More than 5 years  8 I haven't done it before. |
| Question 18 | Could you please tell us the average time per visit (first visit and second visit) for a patient with psoriasis vulgaris or psoriatic arthritis? |
| Response choice 18 | \|  \| first visit \| second visit \| \| --- \| --- \| --- \| \| Psoriasis vulgaris \| X minutes/time \| X minutes/time \| \| Psoriatic arthritis \| X minutes/time \| X minutes/time \| |
| Question 19 | To improve the prognosis of patients with psoriatic arthritis, please select your requests regarding the healthcare environment for optimal diagnosis and treatment from the following options (multiple answers allowed). |
| Response choice 19 | 1 Medical collaboration that allows for early referral if psoriatic arthritis is suspected.  2 Medical collaboration to ensure that patients receive the treatment they need as soon as possible.  3 To be able to make an early diagnosis without a specialist regarding psoriatic arthritis.  4 An opportunity to exchange opinions in cases where the diagnosis of psoriatic arthritis is not clear.  5 An opportunity to exchange opinions on the treatment strategies and therapeutic options in cases where psoriatic arthritis is not clear.  6 Sharing information on patient progress after referral or acceptance.  7 Other requests (please specify)  8 No specific demand |

Supplemental Table 2. Reasons for not performing screening

|  |  | **n** | **Can be diagnosed with physical findings and interview** | **Can be managed with other diagnostic methods** | **Very time consuming** | **Not trustworthy** | **Shortage of facility and staff to manage the tests** |  |
| --- | --- | --- | --- | --- | --- | --- | --- | --- |
| Questionnaire | GP dermatologist | 106 | 43.4 | 12.3 | 17.0 | 0.9 | 27.4 |  |
|  | HP dermatologists | 131 | 41.2 | 13.7 | 33.6 | 3.8 | 16.0 |  |
|  | Rheumatologists | 68 | 51.5 | 27.9 | 32.4 | 2.9 | 19.1 |  |
|  | Orthopedists | 54 | 40.7 | 33.3 | 25.9 | 9.3 | 24.1 |  |
| X-ray | GP dermatologist | 120 | 5.8 | 5.8 | 1.7 | 0.0 | 85.0 |  |
|  | HP dermatologists | 76 | 19.7 | 14.5 | 18.4 | 5.3 | 21.1 |  |
|  | Rheumatologists | 2 | 0.0 | 50.0 | 50.0 | 50.0 | 0.0 |  |
|  | Orthopedists | 7 | 14.3 | 42.9 | 28.6 | 28.6 | 0.0 |  |
| Ultrasound | GP dermatologist | 121 | 5.8 | 5.0 | 1.7 | 3.3 | 83.5 |  |
|  | HP dermatologists | 139 | 12.2 | 15.1 | 22.3 | 1.4 | 39.6 |  |
|  | Rheumatologists | 19 | 36.8 | 31.6 | 26.3 | 15.8 | 52.6 |  |
|  | Orthopedists | 40 | 10.0 | 35.0 | 32.5 | 5.0 | 35.0 |  |
| MRI | GP dermatologist | 123 | 5.7 | 1.6 | 5.7 | 0.8 | 87.0 |  |
|  | HP dermatologists | 117 | 10.3 | 11.1 | 39.3 | 3.4 | 21.4 |  |
|  | Rheumatologists | 20 | 25.0 | 30.0 | 40.0 | 5.0 | 30.0 |  |
|  | Orthopedists | 26 | 23.1 | 19.2 | 46.2 | 7.7 | 30.8 |  |
| Blood test | GP dermatologist | 73 | 21.9 | 8.2 | 8.2 | 6.8 | 46.6 |  |
|  | HP dermatologists | 30 | 23.3 | 6.7 | 26.7 | 6.7 | 26.7 |  |
|  | Rheumatologists | 0 | 0.0 | 0.0 | 0.0 | 0.0 | 0.0 |  |
|  | Orthopedists | 12 | 16.7 | 16.7 | 0.0 | 33.3 | 58.3 |  |

Since the survey was conducted on physicians who did not use questionnaires, imaging (X-ray, ultrasound, and MRI), or blood test, the number of respondents only included two rheumatologists and seven orthopedic surgeons for X-ray analysis, 19 rheumatologists for ultrasound analysis, 20 rheumatologists and 26 orthopedic surgeons for MRI analysis, and zero rheumatologist (no respondent) and 12 orthopedic surgeons for blood test analysis. The respondents were <30.

MRI: magnetic resonance imaging

GP dermatologist: general dermatology practitioners, HP dermatologists: attending dermatologists in hospital
